# Combined Effects of Diatom-Derived Oxylipins on the Sea Urchin *Paracentrotus lividus*

**DOI:** 10.3390/ijms21030719

**Published:** 2020-01-22

**Authors:** Roberta Esposito, Nadia Ruocco, Luisa Albarano, Adrianna Ianora, Loredana Manfra, Giovanni Libralato, Maria Costantini

**Affiliations:** 1 Department of Marine Biotechnology, Stazione Zoologica Anton Dohrn, Villa Comunale, 80121 Napoli, Italy; roberta.esposito@szn.it (R.E.); nadia.ruocco@szn.it (N.R.); luisa.albarano@szn.it (L.A.); ianora@szn.it (A.I.); loredana.manfra@isprambiente.it (L.M.); giovanni.libralato@unina.it (G.L.); 2 Department of Biology, University of Naples Federico II, Complesso Universitario di Monte Sant’Angelo, Via Cinthia 21, 80126 Napoli, Italy; 3 Institute for Environmental Protection and Research (ISPRA), 00144 Rome, Italy

**Keywords:** diatoms, expression level, genes, oxylipins, sea urchin

## Abstract

Oxylipins are diatom-derived secondary metabolites, deriving from the oxidation of polyunsatured fatty acids that are released from cell membranes after cell damage or senescence of these single-celled algae. Previous results revealed harmful toxic effects of polyunsaturated aldehydes (PUAs) and hydroxyacids (HEPEs) on sea urchin *Paracentrotus lividus* embryonic development by testing individual compounds and mixtures of the same chemical group. Here, we investigated the combined effects of these compounds on sea urchin development at the morphological and molecular level for the first time. Our results demonstrated that oxylipin mixtures had stronger effects on sea urchin embryos compared with individual compounds, confirming that PUAs induce malformations and HEPEs cause developmental delay. This harmful effect was also confirmed by molecular analysis. Twelve new genes, involved in stress response and embryonic developmental processes, were isolated from the sea urchin *P. lividus*; these genes were found to be functionally interconnected with 11 genes already identified as a stress response of *P. lividus* embryos to single oxylipins. The expression levels of most of the analyzed genes targeted by oxylipin mixtures were involved in stress, skeletogenesis, development/differentiation, and detoxification processes. This work has important ecological implications, considering that PUAs and HEPEs represent the most abundant oxylipins in bloom-forming diatoms, opening new perspectives in understanding the molecular pathways activated by sea urchins exposed to diatom oxylipins.

## 1. Introduction

Marine organisms are continuously subjected to environmental stressors deriving from human activities [1,2,3,4], climate change [5,6,7,8,9], and toxic metabolites [10,11,12,13,14]. Previous toxicological studies have focused on the exposure of single stress agents but, recently, there is growing interest on the effects of multiple stressors, such as chemical and physical xenobiotics [15,16,17,18,19,20,21,22,23]. However, very few studies are available on natural toxins and on the possible effects of mixtures of these toxins on marine organisms. Oxylipins are a well-known class of diatom secondary metabolites that have been studied for their capability to induce reproductive failure in several marine invertebrates [10,24,25,26,27], including sea urchin *Paracentrotus lividus* [28,29,30,31,32]. Oxylipins represent a group of diverse compounds deriving from the oxidation of polyunsatured fatty acids that are released after cell damage or senescence [11,12,14]. These secondary metabolites include polyunsatured aldehydes (PUAs) and other fatty acid derivatives with hydroperoxy-, hydroxy-, keto-, oxo-, and hydroxy-epoxy functionalities [11,12]. Type and quantity of oxylipins are species-specific [33,34] due to a variety of precursor PUFAs and enzymes [11,12,35,36]. Miralto et al. [10] first isolated and identified PUAs from the bloom-forming diatoms *Thalassiosira rotula*, *Skeletonema marinoi*, and *Pseudo-nitzschia delicatissima* but others were successively identified by Pohnert [35,37,38,39,40,41,42]. Despite PUAs being the first oxylipins identified, almost all diatoms possess LOX pathways for the synthesis of other oxylipins, generically named as non-volatile oxylipins [11,12,43,44]. Oxylipins have multiple biological and biochemical effects inducing the disruption of gametogenesis, gamete functionality, fertilization, embryonic mitosis, and larval fitness and competence in both planktonic and benthic grazers [45]. Previous studies by our group have demonstrated toxigenic effects on sea urchin *P. lividus* embryos exposed to individual polyunsaturated aldehydes (PUAs) and hydroxyacids (HEPEs) [30,31,32] or mixtures of these compounds belonging to the same chemical group [46,47].

In the present work we further explored the toxicity of these natural compounds on *P. lividus* embryonic development by testing PUA and HEPE mixtures. Sea urchin eggs were treated before and after fertilization with PUA and HEPE mixtures at different concentrations, and embryonic development was followed until the pluteus stage to detect possible negative effects at the morphological level. Moreover, we isolated 12 new genes, involved in stress response and embryonic developmental processes, from the sea urchin *P. lividus*. These genes were subjected to interactomic analysis, demonstrating that they were functionally interconnected with 11 genes already described in previous studies investigating the response of *P. lividus* embryos to single oxylipins [30,48,49]. Finally, the expression levels of these 23 genes were followed by real-time qPCR to identify possible gene targets affected by oxylipin mixtures.

## 2. Results

### 2.1. Morphological Effects of PUA/HEPE Mixtures

**Figure 1:**
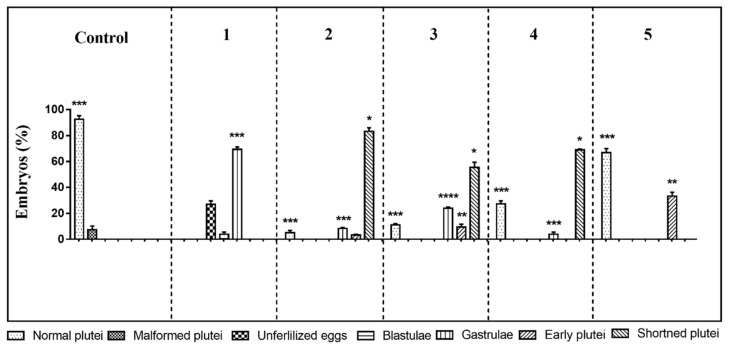
Sea urchin *Paracentrotus lividus* exposed before fertilization to different concentrations of mixtures of polyunsaturated aldehydes (PUA) + hydroxyacids (HEPE). Percentage of normal, malformed, early, delayed, and shortened plutei and gastrulae in controls (embryos grown in the absence of PUA/HEPE mixtures) and treated samples are reported. Five experiments are numbered on the top of the histogram. For the numerical data of the five experiments see also Supplementary Table S1. The statistical significance between different groups is reported as follows: * *p* < 0.05, ** *p* < 0.01, *** *p* < 0.001, **** *p* < 0.0001.

The first experiment was performed at the following concentrations: decadienal 0.5 µM, heptadienal 1.0 µM, and octadienal 1.5 µM and 2.8 µM for the four HEPEs (see also Table 1). At these concentrations, the three PUAs in mixtures produced about 60% malformed embryos [47] and the four HEPEs in mixtures about 40% malformed/delayed embryos [47]. The results showed only 73% of fertilization success; all other eggs underwent first mitotic division, but after 48 h post-fertilization (hpf) all embryos were delayed, with about 3.8% still at the blastula and 69.2% at the gastrula stages (Figure 1; *p*-value < 0.001; see also Table S1). Moreover, embryonic development appeared severely compromised, with embryos showing evident apoptotic processes—mitotic divisions occurred in the blastula, but their phenotypes were irregular and darker inside (see Figure S1D); gastrulae were also abnormal with some cells that were flattened at the vegetal plate but without the invagination of the archenteron (see Figure S1E).

In the second experiment, the oxylipin concentrations were decreased to 0.4, 0.8, and 1.3 µM for decadienal, heptadienal, and octadienal, respectively, and 2.1 μM for HEPEs. This mixture produced no effect on fertilization success and first mitotic division, with a slower delay in development of embryos at 48 hpf. In fact, 83.2% of the embryos reached the pluteus stage but these embryos were shorter than the plutei in control conditions (circulating sea-water without oxylipins; Figure 1 and Figure S1H; *p*-value < 0.05); 8.4% of the gastrulae (Figure S1B; *p*-value < 0.001) and 3.3% of early plutei (Figure 1 and Figure S1F) were non-apoptotic.

In the third experiment, we further decreased the concentrations in the oxylipin mixture to 0.3 µM decadienal, 0.7 µM heptadienal, 1.0 µM octadienal, and 1.8 μM HEPEs. After 48 hpf, about 55.3% of embryos appeared as shortened plutei (Figure 1 and Figure S1H; *p*-value < 0.05) with a low number of delayed embryos at the early pluteus (9.6%; *p*-value < 0.01; Figure S1H) and gastrula stages (24%; *p*-value < 0.0001; Figure S1B).

In the fourth experiment, we only lowered the concentration of the four HEPEs to 1.6 µM and the obtained results were similar to the third experiment, with 60.3% shortened plutei (*p*-value < 0.05) and 5.6% gastrulae (*p*-value < 0.001; Figure 1).

In the last experiment, we tested concentrations of 0.25, 0.5, and 0.75 µM for PUAs (decadienal, heptadienal, and octadienal, respectively) and 1.4 µM for HEPEs (fifth experiment); after 48 hpf, only 33.2% of embryos were delayed at the early pluteus stage (*p*-value < 0.01) and the other embryos (66.8%) were normal plutei, as in the controls (Figure 1 and Figure S1C).

We then extended the exposure time of PUA + HEPE mixtures to 1 week post-fertilization (wpf) to follow the fate of delayed embryos in the mixture with 0.3 µM decadienal, 0.7 µM heptadienal, and 1.0 µM octadienal for PUAs and 1.8 μM for HEPEs. Observations under the light microscope indicated that embryos had reached the pluteus stage but were malformed (degraded arms and apex) because they did not display the characteristic ampoule-like shape, as observed in the controls (Figure S2) [31].

The five experiments were also performed treating sea urchin eggs soon after fertilization at the same mixture concentrations reported above. In these post-fertilization experiments, the results were almost the same, with only a lower percentage of delayed embryos with respect to incubations with oxylipin mixtures added before fertilization (Figure S3 and Table S2).

### 2.2. Network Analysis

**Figure 2:**
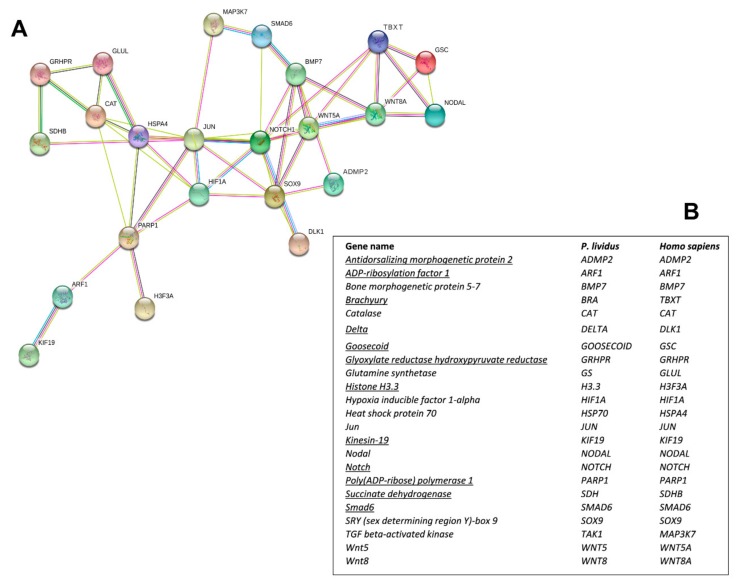
(**A**) Interactomic analysis by STRING (https://string-db.org/). The network graphically displays the relationship between genes. The biological relationships between genes are indicated by different colors. Known interactions: reported by database = light blue and determined experimentally = pink. Expected interactions: gene proximity = green; gene fusion = red; genes with similar pattern = light blue. (**B**) *Homo sapiens* gene names and the corresponding *P. lividus* orthologous genes. The new genes isolated in the present work are underlined.

Interactomic analysis showed that the 12 new genes identified are connected to the following 11 genes, previously analyzed in response to single PUAs and HEPEs treatments: bone morphogenetic protein 5-7 (*BMP5-7*), catalase (*CAT*), glutamine synthetase (*GS*), hypoxia-inducible factor 1-alpha (HIF1A), heat shock protein 70 (*hsp70*), Jun (*Jun*), sex-determining region Y (SRY)-box9 (*Sox9*), TGF beta-activated kinase (*TAK1*), Wnt5 (*Wnt5*), and Wnt8 (*Wnt8*) [30,48,49]. As shown in the network reported in Figure 2A, the genes are correlated as follows:

(1) ADMP2 with BMP5-7 and Sox9; (2) Notch with BMP5-7, Delta, HIF1A, Jun, Smad6, Sox9, and Wnt5; (3) Goosecoid with Brachyury, Nodal, and Wnt8; (4) Brachyury with Goosecoid, Nodal, Notch, Wnt5, and Wnt8; (5) Smad6 with BMP5-7, Notch, and MAPK3/7; (6) GRHPR with CAT, Glut, and SDH; (7) PARP1 with ARF1, CAT, hsp70, Jun, H3.3, and HIF1A; (8) ARF1 with KIF19 and PARP1; (9) SDH with GRHPR and hsp70. The corresponding human orthologous genes are reported in Figure 2B.

### 2.3. Molecular Analyses by Real-Time qPCR

**Figure 3:**
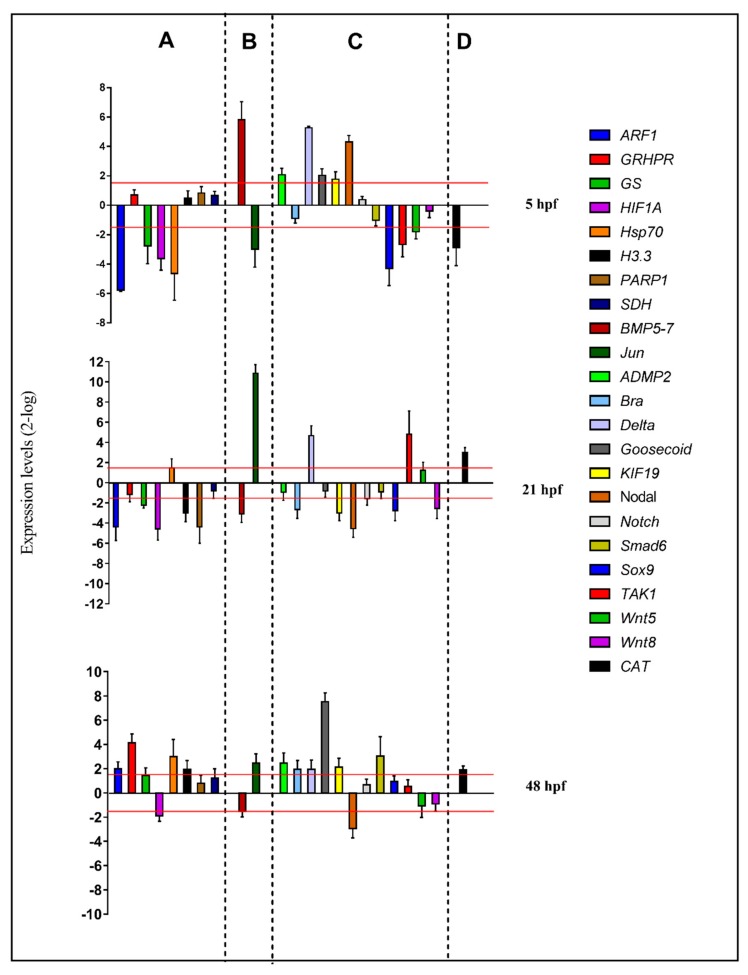
Real-time qPCR at the blastula (5 hpf), gastrula (21 hpf), and pluteus (48 hpf) stages of the sea urchin *P. lividus*. Histograms show the fold-changes of 23 genes involved in different functional processes: stress (**A**), skeletogenesis (**B**), development/differentiation (**C**), and detoxification (**D**). Fold differences greater than ± 1.5 (see red dotted horizontal guidelines at values of +1.5 and −1.5) were considered significant.

For molecular experiments, the expression levels of the 23 functionally interconnected genes were followed by real-time qPCR. Gene expression was detected at the blastula (5 hpf), gastrula (21 hpf), and pluteus (48 hpf) stages, as reported in the histograms in Figure 3 (see also Table S3):

#### 2.3.1. Stress Response Genes

At the blastula stage, four genes were down-regulated with respect to the control: *ARF* (5.8), *GS* (2.8), *HIF1A* (3.6), and *hsp70* (4.6). At the gastrula stage, five genes were targeted by the PUA + HEPE mixture: *ARF* (4.4), *GS* (2.2), *HIF1A* (4.6-), *H3.3* (3.0-), and *PARP1* (4.4), all of which were down-regulated. At the pluteus stage, four genes were up-regulated by the PUA + HEPE mixture: *ARF1* (2.0), *GRHPR* (4.1-), *Hsp70* (3.0), and *H3.3* (2.0). Only the HIF1A gene was down-regulated (2.0).

#### 2.3.2. Skeletogenic Genes

At 5 hpf, *Jun* (2.3) was down-regulated and *BMP5-7* (5.8) was up-regulated by the PUA + HEPE mixture. At the gastrula stage, *Jun* was strongly up-regulated (10.9) and *BMP5-7* (3.1-fold) was down-regulated; then, at the pluteus stage, *Jun* was targeted by PUA + HEPE mixture with an expression level higher than 2.5-fold compared to the control. 

#### 2.3.3. Development/Differentiation Genes

At the blastula stage, six genes were targeted: *sox9* and *TAK1* genes were down-regulated (4.3 and 2.7-fold, respectively), whereas *ADMP2*, *Delta*, *Goosecoid*, and *Nodal* genes were up-regulated (2.1-, 5.3-, 2.0-, and 4.3-fold, respectively). At 21 hpf, *Brachyury*, *KIF19*, *Nodal*, *Sox9*, and *Wnt8* were down-regulated (2.7-, 3.0-, 4.5-, 2.8-, and 2.6-fold, respectively), whereas *TAK1* and *Delta* were up-regulated (4.8- and 4.7-fold, respectively). At 48 hpf, the PUA + HEPE mixture targeted the expression of six genes: *ADMP2*, *Brachyury*, *Delta*, *Goosecoid*, *KIF19*, and *Smad6* were up-regulated (2.5-, 2.0-, 2.0-, 7.5-, 2.1-, and 3.0-fold, respectively). On the contrary, only the Nod*al* gene was down-regulated (3.0-fold).

#### 2.3.4. Detoxification Genes

The only gene followed by real-time qPCR, *CAT*, was targeted by PUA + HEPE mixture at all stages under analysis; in particular, at 5 hpf, this gene showed an increase in its expression level (2.9-fold), whereas at 21 hpf and 48 hpf it was down-regulated (2.0- and 3.0-fold, respectively).

## 3. Discussion

This is the first study describing the negative impact of diatom-derived PUA + HEPE mixtures on sea urchins, considered as model organisms among marine invertebrates. The negative effects of these two classes of oxylipins have been widely studied on the embryonic development of the sea urchin *P. lividus*. Initial studies were performed using single PUAs at low concentrations, demonstrating that decadienal, heptadienal, and octadienal exerted toxigenic effects on embryo development [30]. The strongest effect was induced by decadienal, with heptadienal and octadienal requiring higher concentrations to reach the same effects as decadienal [32]. By contrast, HEPEs induced developmental malformations at much higher concentrations when compared to PUAs. Interestingly, HEPEs also induced a marked developmental delay in sea urchin embryos, which is also evident at higher concentrations, and has not hitherto been reported for PUAs [31]. Our current results indicate that mixtures of these compounds induce a synergistic effect, confirming that PUAs induce malformations and HEPE developmental delay but with a stronger effect with respect to individual compounds [46,47]. The harmful effects of these compounds were also confirmed by molecular analysis, indicating that sea urchins place in motion functionally intercorrelated genes to counteract negative effects against these natural toxins [48,49,50].

Our morphological results demonstrate a synergistic effect of the seven oxylipins in mixtures. More specifically, PUA and HEPE mixtures induce a dose-dependent delay in sea urchin development. Even if PUAs in individual tests seem to be stronger than HEPEs, when in mixtures the effects of HEPEs seem to dilute those of PUAs. In fact, in the first 48 hpf, oxylipin mixtures only induced delay of sea urchin embryos and no malformed embryos were detected. Malformations of *P. lividus* embryos (usually induced by individual PUAs or in mixtures of PUAs) were visible only after one-week post-fertilization. These results indicate that PUAs + HEPEs induce delay but not arrest of embryonic development. These findings can be explained in light of previous results demonstrating a reversible effect for individual PUAs and irreversible effects for HEPEs. *P. lividus* embryos can recover after exposure to PUAs [30] but not HEPEs [31]. Our morphological results are also linked to the molecular response of sea urchin embryos to PUAs that occurred later (at the pluteus stage) compared to HEPEs (at the blastula stage). Taken together, these results indicate that the delay observed in the early development of sea urchins may be due to HEPEs, which act in an irreversible way and precociously on embryos, targeting many genes involved in skeletogenesis and development/differentiation processes already at the blastula stage. PUA-induced malformations were detectable only one week post fertilization, with gene expression mainly affected later at the pluteus stage.

**Figure 4:**
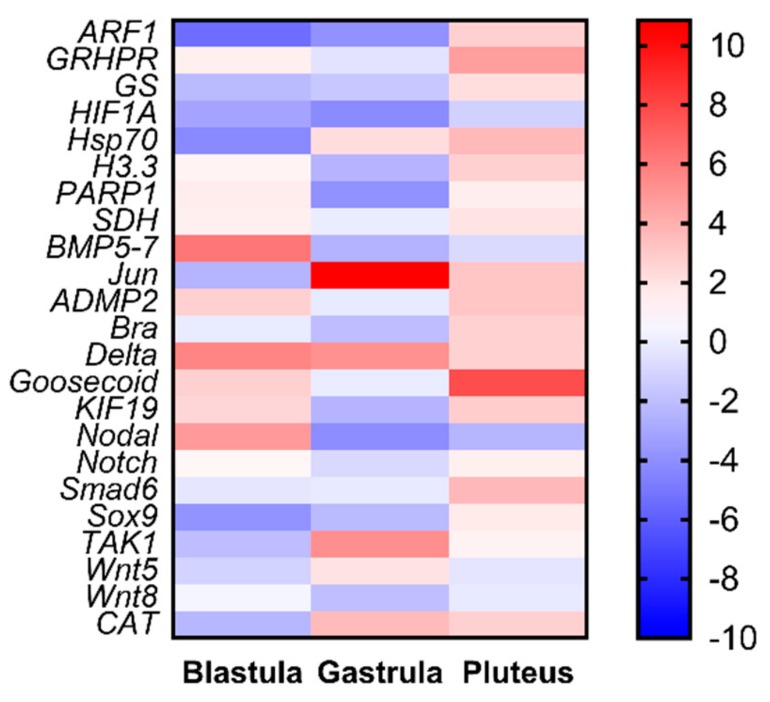
Heatmaps (Heatmapper available at www.heatmapper.ca) showing the expression profiles and hierarchical clustering of 23 genes analyzed by real-time qPCR in *P. lividus* embryos at the three different developmental stages (blastula, gastrula, and pluteus) treated with mixtures decadienal 0.3 μM, heptadienal 0.7 μM, and octadienal 1.0 μM, and HEPEs 1.6 μM. Colour code: red, up-regulated genes respect to the control (embryos grown without PUAs + HEPEs mixtures); blue, down-regulated genes.

Our study also provides new information on the molecular effects of oxylipins on sea urchin embryos. Even if *P. lividus* represents a well-established model organism for ecotoxicological studies, its genome is still not available. Hence, the availability of 12 new genes provides a better understanding on the molecular mechanisms of response in sea urchins to toxins. An important outcome of this work consists in the fact that these new genes are not only functionally intercorrelated with one another, but also with another 11 genes previously analyzed in response to tests on individual PUAs and HEPEs. PUA + HEPE mixtures are able to affect the expression level of most of the analyzed genes, with the only exception of *SDH*, *Notch*, and *Wnt5*. These genes were involved in stress, skeletogenesis, development and differentiation, and detoxification processes. Furthermore, PUAs + HEPEs targeted genes in all three developmental stages analyzed (as summarized in the heat map reported in Figure 4), mainly down-regulating genes at the early gastrula stage and up-regulating these genes at the pluteus stage. It is worth noting that the new genes (mainly those involved in differentiation and developmental processes) isolated in this work have never been studied before in response to environmental stress.

To date, no data were available on the combined effects of natural toxins on the sea urchin *P. lividus*. Several studies focused on the exposure of sea urchins to heavy metal mixtures and combinations with other stressors [15,16,17], as the anthropogenic release into the environment of these chemicals represents a serious risk for normal physiological processes of exposed biota. Moreover, many studies have also explored how ocean acidification and warming may both impact the reproductive success and larval fitness of sea urchins [18,19,20,21,22,23].

This study can be considered a step forward in the ecotoxicological assessment of natural toxins on these organisms. Given the importance of diatom blooms in nutrient-rich aquatic environments, our findings acquire interesting ecological implications. In fact, hydroxyacids represent one of the most common classes of oxylipins produced by some diatoms, much more common than the better-known PUAs [13,35,39,51,52]. Both PUA-producing and non-producing diatoms are able to negatively affect hatching success in copepods, with induction of apoptosis in newly hatched nauplii [12].

In a previous study, we demonstrated that the concentrations tested in this study are well within the significant range for affecting growth and performance of sea urchins during bloom conditions [31]. In their natural habitats sea urchins and their larvae may come in contact with diatom PUAs in the field at the end of a bloom [53]. In fact, in aquatic ecosystems, a considerable proportion of the primary production from phytoplankton bloom sinks to the sediment [54,55]. Because sea urchins are browsing animals that eat phytoplankton and organic matter in the sand or mud, they may accumulate oxylipins through feeding or be exposed to high local concentrations of these compounds that may affect growth performance, as already demonstrated for copepods exposed to PUAs [45]. Very recently, Russo et al. [56] reported on oxylipin production rates in natural planktonic diatom communities in the Gulf of Naples, from which sea urchins were collected in the present study. These authors provided the first piece of evidence that the oxylipins analysed from natural phytoplankton communities derive mostly from diatoms. High phytoplankton abundance was observed from April to July, whereas low values were detected in March and November. Overall, diatoms dominated phytoplankton assemblages during peaks observed in late spring, summer, and September–October, corresponding to the reproductive period of the sea urchin *P. lividus*. Not only planktonic diatoms, but also benthic diatoms from the Gulf of Naples have been shown to produce oxylipins [57,58,59,60], with dramatic effects on benthic grazers [24], including the sea urchin *P. lividus*, indicating that potentially all diatoms can negatively impact reproductive success and development in sea urchins.

More generally, this study on multiple stressors can also help predict and understand their effects through the study of the molecular pathways activated in response to stressors, allowing organisms to counteract deleterious consequences and irreversible damage.

## 4. Materials and Methods

### 4.1. Ethics Statement

*Paracentrotus lividus* (Lamarck) were collected from a site in the Bay of Naples that is not privately owned or protected in any way, according to Italian legislation (DPR 1639/68, 19 September 1980, confirmed on 10 January 2000). Field studies did not include endangered or protected species. All experimental procedures on animals were in compliance with the guidelines of the European Union (directive 2010/63/EU).

### 4.2. Gamete Collection, Egg Incubation with Mixtures of PUAs and HEPEs, Embryo Cultures, and Morphological Analyses

Adult sea urchin *P. lividus* were collected in the Gulf of Naples during their reproductive period by scuba diving and were stored in tanks with circulating sea-water (FSW) until testing. To induce gamete emission, sea urchins were shaken or injected with 2 M KCl through the peribuccal membrane. Eggs were washed with filtered sea water and kept in FSW until use. Dry sperm was collected and kept undiluted at +4 °C until use. Experiments were performed treating eggs with the seven oxylipins: 2-trans,4-trans-decadienal (Sigma-Aldrich, St. Louis, MO, USA), 2-trans,4-trans-heptadienal (Sigma-Aldrich), 2-trans,4-trans-octadienal (Sigma-Aldrich), 5-hydroxy-6E,8Z,11Z,14Z,17Z-eicosapentaenoic acid (5-HEPE; Cayman Chemical, Ann Arbor, MI, USA; purity ≥98%), 9-hydroxy-5Z,7E,11Z,14Z,17Z-eicosapentaenoic acid (9-HEPE; Cayman Chemical, Ann Arbor, MI, USA; purity ≥98%), 11-hydroxy-5Z,8Z,12E,14Z,17Z-eicosapentaenoic acid (11-HEPE; Cayman Chemical, Ann Arbor, MI, USA; purity ≥98%), and 15-hydroxy-5Z,8Z,11Z,13E,17Z-eicosapentaenoic acid (15-HEPE; Cayman Chemical, Ann Arbor, MI, USA; purity ≥98%). Five experiments were performed adding the oxylipins 10 min before and 10 min after fertilization (with the exception of the fourth and fifth experiments performed by only treating eggs before fertilization) at different concentrations, as reported in Table 1.

Eggs were fertilized using sperm-to-egg ratios of 100:1 for both controls and treatments. Experiments were conducted in triplicate, collecting eggs from three different females. A control experiment was conducted fertilizing eggs in FSW without PUA/HEPE mixtures. To evaluate the effects of mixture treatments, three different endpoints were checked: (i) fertilization success, (ii) first cleavage, and (iii) pluteus stage (48 hpf). At 48 hpf, controls and treated embryos were fixed with glutaraldehyde (1% in FSW) and observed under the light microscope (Zeiss Axiovert 135TV, Carl Zeiss, Jena, Germany) to assess the percentage of normal and malformed plutei.

### 4.3. RNA Extraction and cDNA Synthesis

About 5000 eggs in 12 mL of FSW were treated with mixtures of three PUAs (0.3 μM decadienal, 0.7 μM heptadienal, 1.0 μM octadienal) and four hydroxyacids (5-, 9-, 11-, 15-HEPEs 1.8 μM) for 10 min and then fertilized. Samples were then collected at blastula, early gastrula, and pluteus, corresponding to 5, 21, and 48 hpf, respectively, and centrifuged at 3500 relative centrifugal force for 15 min in a swing out rotor at 4 °C. The pellet was washed with phosphate-buffered saline and then frozen in liquid nitrogen and kept at −80 °C until use. Total RNA was extracted using Aurum Total RNA Mini Kit (Bio-Rad, Hercules, California, USA), according to [61]. The amount of total RNA extracted was estimated by the absorbance at 260 nm and the purity by 260/280 and 260/230 nm ratios, using a NanoDrop spectrophotometer (ND-1000 UV-VIS Spectrophotometer; NanoDrop Technologies, Wilmington, DE, USA). The integrity of RNA was evaluated observing the rRNA subunits (28S and 18S) on agarose gel electrophoresis. For each sample, 1000 ng of total RNA was retrotranscribed with an iScript cDNA Synthesis kit (Bio-Rad, Milan, Italy), following the manufacturer’s instructions. Experiments were conducted in triplicate using different egg groups collected from four individuals.

### 4.4. Gene Isolation and Interactomic Analysis

The sequences of 12 new genes were retrieved from the transcriptome of the sea urchin *P. lividus* deposited in the SRA database (Sequence Read Archive, available at https://www.ncbi.nlm.nih.gov/sra, accession number PRJNA495004) [60] and from the Taxonomy Broswer (available at https://www.ncbi.nlm.nih.gov/Taxonomy/Browser/wwwtax.cgi). For each gene, specific primers were designed on the nucleotide sequences (see Table 2).

PCR fragments were purified from agarose gel using the QIAquick Gel Extraction kit (Qiagen, Milan, Italy), and the specificity of the PCR product was checked by DNA sequencing. Five serial dilutions were set up to determine Ct values and PCR efficiencies for all primer pairs (for real-time qPCR conditions, see below). The efficiency of each primer pair was calculated by E = [10^(-1/slope)-1]*100 (Table 2). Standard curves were generated for each oligonucleotide pair using Ct values versus the logarithm of each dilution factor.

The network analysis was performed by STRING [62], an online database of known and predicted protein–protein interactions, to identify relationships on the basis of associated functions and data mining from experimental studies reported in the literature. Because sea urchin genes are not annotated in the STRING database, we used the name of the human orthologues to search for *P. lividus* genes (see Figure 1). The biological functions for these 12 genes are reported in Table S4.

### 4.5. Real-Time qPCR

Molecular analyses were performed on specimens treated with PUA/HEPE mixtures at 1.6 µM for HEPEs and 0.3 µM decadienal, 0.7 µM heptadienal, and 1 µM octadienal. Samples were then collected at different developmental stages after fertilization, corresponding to early blastula (5 hpf), late gastrula (21 hpf), and pluteus (48 hpf). Undiluted cDNA was used as a template in a reaction containing a final concentration of 0.3 mM for each primer and 1× FastStart SYBR Green master mix (total volume of 10 μL) (Applied Biosystems, Monza, Italy). PCR amplifications were performed in a ViiATM7 Real-Time PCR System (Applied Biosystems, Monza, Italy) thermal cycler using the following thermal profile: 95 °C for 10 min, one cycle for cDNA denaturation; 95 °C for 15 s and 60 °C for 1 min, 40 cycles for amplification; 72 °C for 5 min, one cycle for final elongation; one cycle for melting curve analysis (from 60 to 95 °C) to verify the presence of a single product. Each assay included a no-template control for each primer pair. To capture intra-assay variability, all real-time qPCR reactions were carried out in triplicate. Fluorescence was measured using the ViiATM7 software (Applied Biosystems, Monza, Italy). The expression of each gene was analyzed and internally normalized against ubiquitin [29] and 18S rRNA [63,64] using REST software (Relative Expression Software Tool, Weihenstephan, Germany) on the basis of the Pfaffl method [65,66]. Relative expression ratios above 1.5 were considered significant (see Table S3).

## Figures and Tables

**Table 1:** Concentrations in micromolar (μM) of the seven oxylipins in the five experiments.

Experiment	Concentration (μM)						
	Decadienal	Heptadienal	Octadienal	5-HEPE	9-HEPE	11-HEPE	15-HEPE
1	0.5	1.0	1.5	2.8	2.8	2.8	2.8
2	0.4	0.8	1.3	2.1	2.1	2.1	2.1
3	0.3	0.7	1.0	1.8	1.8	1.8	1.8
4	0.3	0.7	1.0	1.6	1.6	1.6	1.6
5	0.25	0.5	0.75	1.4	1.4	1.4	1.4

**Table 2:** Gene name, acronym, accession numbers, primer names, primer sequences, primer efficiency, and amplicon lengths of PCR fragments. Accession number indicated with * derived from the transcriptome (accession number PRJNA495004)

Gene Names	Acronym	Accession Number	Primer	Sequence(5′→3′)	Amplicon Length (bp)	Efficiency (%)
*Antidorsalizing morphogenetic*	*ADMP2*	KT276376	ADMP2_F1	CGAGCAAGAGGCTCCTCC	161	96
*protein 2*			ADMP2_R1	CTTTGGCAACGCCGACAAAC		
*ADP-ribosylation factor 1*	*ARF1*	SUB2817153 *	ADP_F2	CTGGTGCATCAGTAAACTAC	256	100
			ADP_R2	CGTCGTAAGAGCAGGAACG		
*Brachyury*	*Bra*	AJ419790	BRA_F1	CCGCGAACTCGACCGAAC	178	94
			BRA_R1	CAACCCGACAGAGCTCCC		
*Delta*	*Delta*	DQ536193	DELTA_F1	CTGCATGCGAGATGTCATG	178	101
			DELTA_R1	GCAACGTAGATGACCAGCTG		
*Goosecoid*	*Goosecoid*	HM449798	Goosecoid_F1	GCGCGCATGTCCAGACTG	161	98
			Goosecoid_R1	GTCCATCTCAAAGATCAGAGC		
*Glyoxylate reductase*	*GRHPR*	SUB2817153 *	GLR_F1	GGCTCACAACAGATGAAGTAG	171	100
*hydroxypyruvate reductase*			GLR_R1	CTTGGCGTATCTTCGTTCTC		
*Histone H3.3*	*H3.3*	X76081	H3.3_F1	GCTGCTATTGGTGCTCTG	156	93
			H3.3_R1	GATTGATCAGGATGGTCACG		
*Kinesin-19*	*KIF19*	SUB2817153 *	KIF19_F1	CGCAGACTCATCACAGAGC	176	106
			KIF19_R1	GCCTTGGCCACCTTCATGC		
*Notch*	*Notch*	JQ861276	Notch_F1	CGAGAGGGAGGTGGATAGC	188	94
			Notch_R1	GGGTTGCTCACACAGTGG		
*Poly(ADP-ribose)*	*PARP-1*	SUB2817153 *	pADP_F1	GGCAATGATGCTGTCTGTAG	184	101
*polymerase 2*			pADP_R1	CAGTTACAGCACTGGTTTCATC		
*Succinate dehydrogenase*	*SDH*	SUB2817153 *	SDH_F2	GGTGAGTAGGAAATTCACAGTG	210	102
*assembly factor mitochondrial*			SDH_R2	CTCACGTCGGAATGTCTTCG		
*Smad6*	*Smad6*	HM449802	Smad6_F1	CGAGACCGACCTCAAAGC	134	105
			Smad6_R1	GTTCCCCTTTCGAGATGG		

## Supplementary Materials

Supplementary Materials can be found at https://www.mdpi.com/1422-0067/21/3/719/s1.
