# Supplementary material for: Combined Effects of Diatom-Derived Oxylipins on the Sea Urchin Paracentrotus lividus
Source: Int J Mol Sci. 2020 Jan 22;21(3):719. doi: 10.3390/ijms21030719 (PMC7036778; doi:10.3390/ijms21030719)
Supplement: Supplementary file 1 [file ijms-21-00719-s001.pdf]

Supplementary Material

Combined effects of diatom-derived oxylipins on the sea urchin *Paracentrotus lividus*

Roberta Esposito[1], Nadia Ruocco1[,#], Luisa Albarano[1,2,#], Adrianna Ianora[1], Loredana Manfra[1,3], Giovanni Libralato[1,2], Maria Costantini[1,*]

[1]Department of Marine Biotechnology, Stazione Zoologica Anton Dohrn, Villa Comunale, 80121 Napoli; R.E. roberta.esposito@szn.it; N.R. nadia.ruocco@szn.it; L.A. luisa.albarano@szn.it; A.I. ianora@szn.it
[2]Department of Biology, University of Naples Federico II, Complesso Universitario di Monte Sant'Angelo, Via Cinthia 21, 80126, Napoli, Italy; G.L. Giovanni.libralato@unina.it
[3]Institute for Environmental Protection and Research (ISPRA), 00144 Rome, Italy; L.M. loredana.manfra@isprambiente.it

# These authors contributed equally to this work

* Correspondence: M.C., email: maria.costantini@szn.it; Tel.: +39 08158333285

Supplementary Figure S1. Photos (taken with Zeiss Axiovert 135TV microscope, 10x / 0.30, magnification / numerical aperture) of controls at the blastula (A, 5 hpf), gastrula (B, 21 hpf) and pluteus (C, 48 hpf) stage (embryos in sea water without PUA/HEPE mixtures), D) apoptotic blastulae, E) apoptotic gastrulae, F) early plutei, G-H) shortened plutei. Scale Bar: 50 µm.

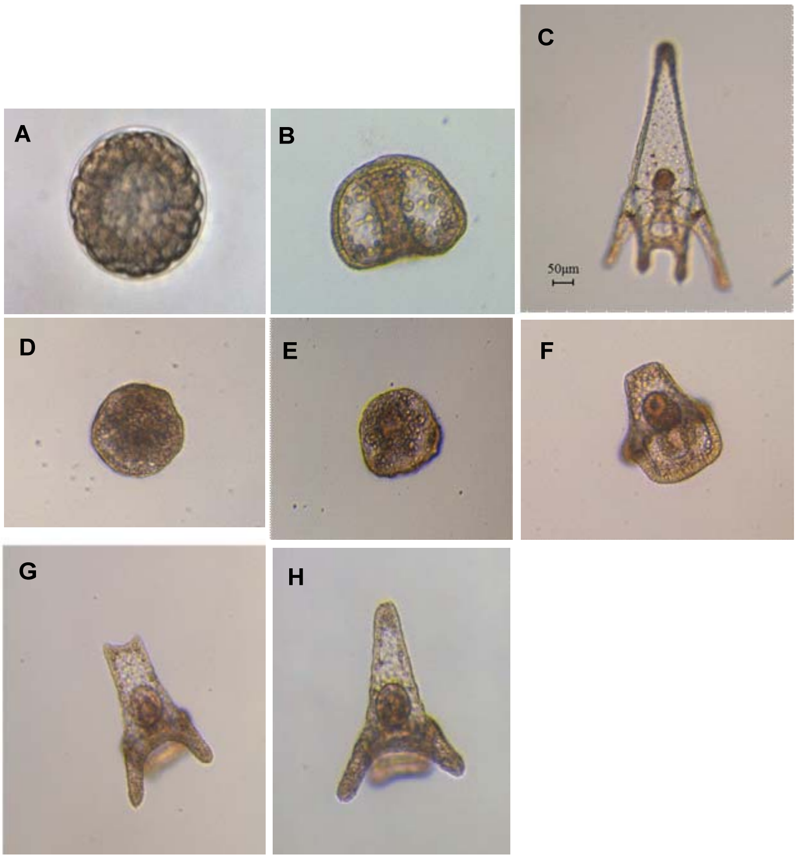

Supplementary Figure S2. Photos (taken with Zeiss Axiovert 135TV microscope, 10x / 0.30, magnification / numerical aperture) of A-B) controls (embryos in sea water without PUA/HEPE mixtures) at 1 wpf, C-E) abnormal embryos after incubation with PUA/HEPE mixtures. Scale Bar: 50 μm.

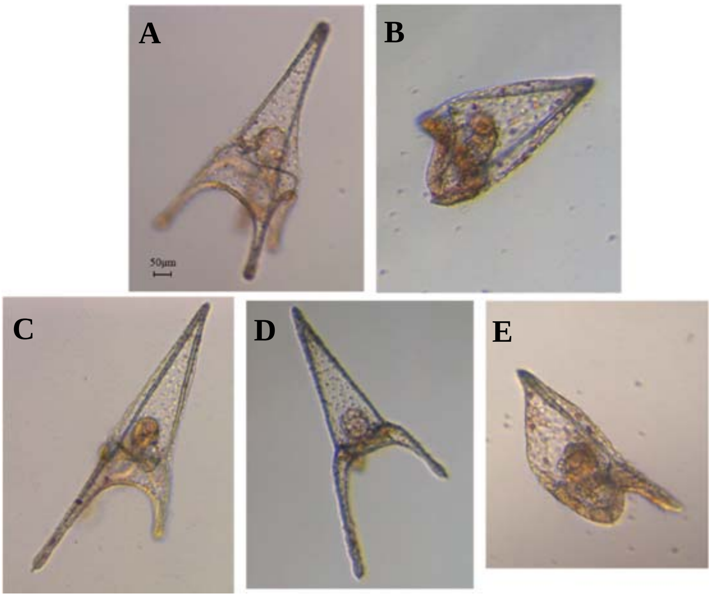

Supplementary Figure S3. Percentage of normal, malformed, early plutei, delayed and shortened plutei and gastrulae in controls (embryos grown in absence of PUA/HEPE mixtures) and treated samples after fertilization. Three experiments with different concentrations (see Materials and Methods and Supplementary Table S2) were numbered on the top of the histogram. The statistical significance between different groups was performed by *GraphPad Prism version 7* (GraphPad Software, La Jolla, California, USA, www.graphpad.com). Student's *t*-tests (* $p<0.05$, ** $p<0.01$, *** $p<0.001$, **** $p<0.0001$).

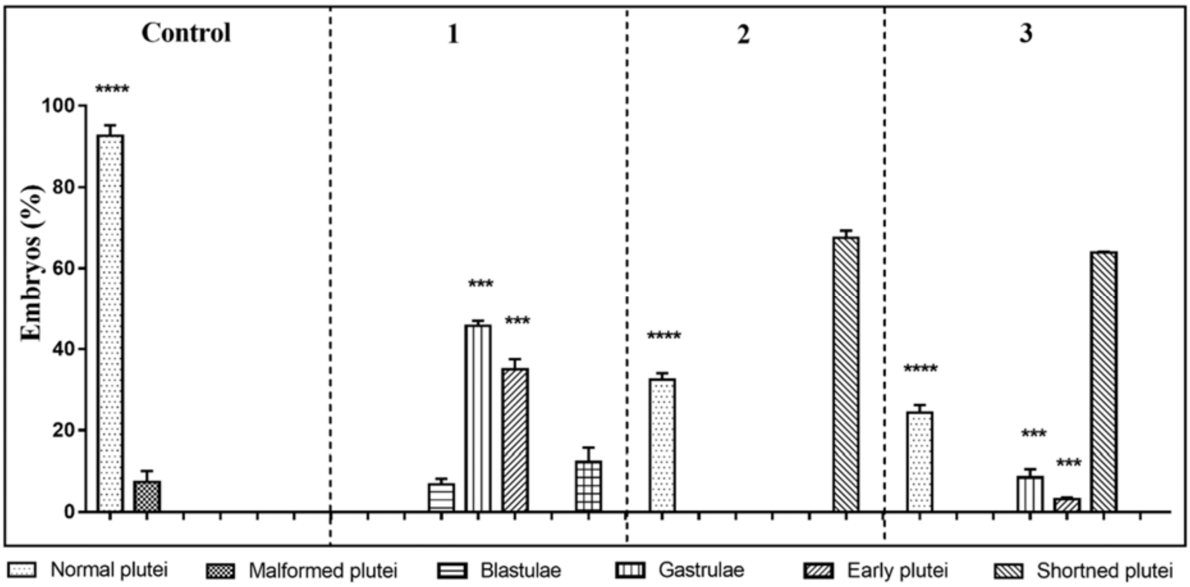

Supplementary Table S1. Percentage of normal plutei, malformed plutei, blastulae, gastrulae, early plutei, shortened plutei in controls (embryos grown in absence of PUA/HEPE mixtures) and from sea urchin *P. lividus* exposed before fertilization to different concentrations of mixtures of PUA+HEPE in the five experiments.

**Experiment**	**Normal plutei**	**Malformed plutei**	**Unfertilized eggs**	**Blastulae**	**Gastrulae**	**Early plutei**	**Shortened plutei**
Control	91.4	8.6					
1			27.0	3.8	69.2		
2	5.1				8.4	3.3	83.2
3	11.1				24.0	9.6	55.3
4	34.1				5.6		60.3
5	66.8					33.2	

Supplementary Table S2. Percentage of normal, malformed, early plutei, delayed and shortened plutei and gastrulae in controls (embryos grown in absence of PUA/HEPE mixtures) and treated samples after fertilization in the three experiments with different concentrations.

Experiment						
	**Normal plutei**	**Malformed plutei**	**Blastulae**	**Gastrulae**	**Early plutei**	**Shortened plutei**
Control	92.6	7.8				
1			6.8	45.8	35.0	12.4
2	32.4					67.6
3	53.5			8.5	3.2	34.8

Supplementary Table S3. Fold changes reported for each gene analysed by Real Time qPCR at 5, 21 and 48 hpf. Up-regulated genes= red, down-regulated genes= blue.

	5 hpf	21 hpf	48 hpf
Stress			
ARF1	-5.8	-4.4	2.0
GRHPR	0.7	-1.2	4.1
GS	-2.8	-2.2	1.4
HIF1A	-3.6	-4.6	-1.9
Hsp70	-4.6	1.5	3.0
H3.3	0.5	-3.0	2.0
PARP1	0.8	-4.4	0.8
SDH	0.7	-0.8	1.2
Skeletogenesis			
BMP5-7	5.8	-3.1	-1.5
Jun	-3.0	10.9	2.5
Development/Differentiation			
ADMP2	2.1	-0.9	2.5
Brachyury	-0.9	-2.7	2.0
Delta	5.3	4.7	1.9
Goosecoid	2.0	-0.8	7.5
KIF19	1.8	-3.0	2.1
Nodal	4.3	-4.5	-3.0
Notch	0.4	-1.6	0.7
Smad6	-1.0	-0.9	3.0
Sox9	-4.3	-2.8	0.9
TAK1	-2.7	4.8	0.5
Wnt5	-1.8	1.2	-1.1
Wnt8	-0.4	-2.6	-0.9
Detoxification			
CAT	-2.9	3.0	2.0

Supplementary Table S4. Names, acronym, functions and references of the twelve new genes isolated in the present work.

Gene name	Acronym	Function	References
Antidorsalizing morphogenetic protein 2	ADMP2	This gene encodes for a protein positively regulated by the *BMP* signal. Moreover, it is expressed in dorsal-ventral ectoderm and it is required for doral-lateral ectoderm specification.	[1]
ADP-ribosylation factor 1	ARF1	An enzyme that catalyzes the trasfer of ADP-rybose from NAD+ to proteins causing their inactivation.	[2-4]
Brachyury	Bra	Trascription factor that plays a kay role in the differentiation of mesoderm and endoderm.	[5-7]
Delta	Delta	This protein, binding to the *Nocht* receptor on the cell surface, causes the splitting of intracellular domain (Nic), which enters the nucleus and activates the trascription of target genes involved in mesoderm formation.	[8-11]
Goosecoid	Goosecoid	Trascription factor able to induce the expression of two genes, *FOXA* and *Bra*, involved in stomodeal formation. It also inhibits the ciliary band formation and the dorsal genes expression.	[12]
Glyoxylate reductase hydroxypyruvate reductase	GRHPR	Member of oxidoreductase family that plays a kay role in the reaction of hydroxypiruvate formation starting from D-glycerate.	[13]
Histone H3.3	H3.3	Basic protein involved in the chromatin structure and in the gene expression regulation.	[14,15]
Kinesin-19	KIF19	Transporter protein of organelle and protein complex to specific destinations depending to ATP and microtubules.	[16-22]
Notch	Notch	Protein involved in endomesoderm segregation and in the specific mesoderm genes activation.	[9,23]
Poly(ADP-ribose) polymerase 2	PARP1	Activation of *PARP1* causes the release of *AIF*, mitochondrial oxidoreductase that induces apoptosis.	[24]
Succinate dehydrogenase assembly factor mitochondrial	SDH	Enzyme involved in the Krebs cycle and in the electron transport chain.	[25]
Smad6	Smad6	This trascription factor, firstly expressed in mesenchymal blastula, is essential for the specification of embryo dorsal side.	[12,26]

References

1. Lapraz, F.; Haillot, E.; Lepage, T. A deuterostome origin of the Spemann organizer suggested by Nodal and ADMP2 functions in Echinoderms. *Nat. Commun.* **2015**, *6*, 8927. doi: 10.1038/ncomms9434.
2. Beane, W.S.; Voronina, E.; Wessel, G.M.; McClay, D.R. Lineage-specific expansions provide genomic complexity among sea urchin GTPases. *Dev. Biol.* **2006**, *300*, 165-179. doi: 10.1016/j.ydbio.2006.08.046.
3. Kahn, R.A.; Volpicelli-Daley, L.; Bowzard, B.; Shrivastava-Ranian, P.; Li, Y.; Zhou, C.; Cunningham, L. Arf family GTPases: roles in membrane traffic and microtubule dynamics. *Biochem. Soc. Trans.* **2005**, *33*, 1269-1272. doi:10.1042/BST20051269.
4. Wennerberg, K.; Rossman, K.L.; Der, C.J. The Ras superfamily at a glance. *J. Cell Sci.* **2005**, *118*, 843-6. doi:10.1242/jcs.01660.
5. Croce, J.; Lhomond, G.; Gache, C. Expression pattern of *Brachyury* in the embryo of the sea urchin *Paracentrotus lividus*. *Dev. Genes Evol.* **2001**, *212*, 617-619. doi: 10.1007/s00427-001-0200-5
6. Harada, Y.; Yasuo, H.; Satoh, N. A sea urchin homologue of the chordate *Brachyury* (T) gene is expressed in the secondary mesenchyme founder cells. *Development* **1995**, *121*, 2747-2754.
7. Peterson, K.J.; Harada, Y.; Cameron, R.A.; Davidson, E.H. Expression pattern of *Brachyury* and *Notch* in the sea urchin: comparative implications for the origins of mesoderm in the basal deuterostomes. *Dev. Biol.* **1999b**, *207*, 419-431. doi.org/10.1006/dbio.1998.9177

8. Materna, S.C.; Nam, J.; Davidson, E.H. High accuracy, high resolution prevalence measurement for the majority of locally expressed regulatory genes in early sea urchin development. *Gene Expr. Patterns.* **2010**, *10*, 177-184. doi: 10.1016/j.gep.2010.04.002
9. Materna, S.C.; Davidson E.H. A comprehensive analysis of Delta signaling in pre-gastrular sea urchin embryos. *Dev. Biol.* **2012**, *364*, 77-87. doi: 10.1016/j.ydbio.2012.01.017
10. Revilla-i-Domingo, R.; Minokawa, T.; Davidson, E.H. R11: a cis-regulatory node of the sea urchin embryo gene network that controls early expression of Sp Delta in micromeres. *Dev. Biol.* **2004**, *274*, 438-451. doi: 10.1016/j.ydbio.2004.07.008
11. Smith, J.; Davidson, E.H. Gene regulatory network subcircuit controlling a dynamic spatial pattern of signaling in the sea urchin embryo. *Proc. Natl. Acad. Sci.* **2008**, *105*, 20089-20094. doi: 10.1073/pnas.0806442105
12. Saudemont, A.; Haillot, E.; Mekpoh, F.; Bessodes, N.; Quirin, M.; Lapraz, F.; Duboc, V.; Röttinger, E.; Ranger, R.; Oisel, A.; Besnardeau, L.; Wincker, P.; Lepage, T. Ancestral regulatory circuits governing ectoderm patterning downstream of *Nodal* and *BMP2/4* revealed by gene regulatory network analysis in an echinoderm. *Plos Genet.* **2010**, *6*, e1001259. doi: 10.1371/journal.pgen.1001259
13. Lassalle, L., Engilberge, S., Madern, D., Vauclare, P., Franzetti, B., Girard, E. New insights into the mechanism of substrates trafficking in Glyoxylate/Hydroxypyruvate reductases. *Sci. Rep.* 2016, *6*, 23879. doi: 10.1038/srep20629
14. Mancini, P.; Dentici, M.; Aniello, F.; Branno, M.; Piscopo, M.; Pulcrano, G.; Fucci, L. The replacement *H3.3* histone gene in *Paracentrotus lividus* sea urchin: structure and regulatory elements. *Biochim. Biophys. Acta.* **2001**, *1519*, 39-45. doi: 10.1016/s0167-4781(01)00205-6

15. Fucci, L.; Aniello, F.; Branno, M.; Biffali, E., Geraci, G. Isolation of a new *H3.3* histone variant cDNA of *Paracentrotus lividus* sea urchin: sequence and embryonic expression. *Biochim. Biophys. Acta.* **1994**, *1219*, 539-542. doi: 10.1016/0167-4781(94)90084-1

16. Bi, G.Q.; Morris, R.L.; Liao, G.; Alderton, J.M.; Scholey, J.M.; Steinhardt, R.A. Kinesin- and myosyn-driven steps of vesicle recruitment for $Ca2^{+}$-regulated exocytosis. *J. Cell Biol.* **1997**, *138*, 999-1008. doi: 10.1083/jcb.138.5.999

17. Wright, B.D.; Henson, J.H.; Wedaman, K.P.; Willy, P.J.; Morand, J.N.; Scholey, J.M. Subcellular localization and sequence of sea urchin kinesin heavy chain: evidence for its association with membranes in the mitotic apparatus and interphase cytoplasm. *J. Cell Biol.* **1991**, *113*, 817-833. doi: 10.1083/jcb.113.4.817

18. Chui, K.K.; Rogers; G.C.; Kashina, A.M., Wedaman, K.P., Sharp, D.J., Nguyen, D.T.; Wilt, F.; Scholey, J.M. Roles of two homotetrameric kinesins in sea urchin embryonic cell division. *J. Biol. Chem.* **2000**, *275*, 38005-38011. doi:10.1074/jbc.M005948200

19. Rogers, G.C.; Chui, K.K.; Lee, E.W.; Wedaman, K.P.; Sharp, D.J.; Holland, G.; Morris, R.L.; Scholey, J.M. A kinesin-related protein, KRP(180), positions prometaphase spindle poles during early sea urchin embryonic cell division. *J. Cell Biol.* **2000**, *50*, 499-512. doi: 10.1083/jcb.150.3.499

20. Sharp, D.J.; Rogers, G.C.; Scholey, J.M. Roles of motor proteins in building microtubule-based structures: a basic principle of cellular design. *Biochim. Biophys. Acta.* **2000**, *1496*, 128-141. doi: 10.1016/s0167-4889(00)00014-8

21. Morris, R.L.; Scholey, J.M. Heterotrimeric kinesin-II is required for the assembly of motile 9+2 ciliary axonemes on sea urchin embryos. *J. Cell Biol.* **1997**, *138*, 1009-1022. doi: 10.1083/jcb.138.5.1009

22. Morris, R.L.; Hoffaman, M.P.; Obar, R.A.; McCafferty, S.S.; Gibbons, I.R.; Leone, A.D.; Cool, J.; Allgood, E.L.; Musante, A.M.; Judkins K.M.; Rossetti, B.J.; Rawson, A.P.; Burgess, D.R. Analysis of cytoskeletal and motility proteins in the sea urchin genome assembly. *Dev. Biol.* **2006**, *300*, 219-237. doi: 10.1016/j.ydbio.2006.08.052
23. Warner, J.F.; McClay, D.R. Left-right asymmetry in the sea urchin. *Genesis* **2014**, *52*, 481-487. doi.org/10.1002/dvg.22752
24. Yu, S.W.; Andrabi, S.A.; Wang, H.; Kim, N.S.; Poirier, G.G.; Dawson, T.M.; Dawson, V.L. Apoptosis-inducing factor mediates poly(ADP-ribose) (PAR) polymer-induced cell death. *Proc. Natl. Acad. Sci.* **2006**, *103*, 18314-18319. doi: 10.1073/pnas.0606528103
25. Rasheed, M.R.H.A.; Tarjan, G. Succinate dehydrogenase complex: an updated review. *Arch. Pathol. Lab. Med.* **2018**, *142*, 1564-1570. doi: 10.5858/arpa.2017-0285-RS
26. Ku, M.C.; Stewart, S.; Hata, A. Poly(ADP-ribose) polymerase 1 interacts with OAZ and regulated BMP-target genes. *Biochem. Biophys. Res. Commun.* **2003**, *311*, 702-707. doi: 10.1016/j.bbrc.2003.10.053
